# The effects of deprivation and relative deprivation on self-reported morbidity in England: an area-level ecological study

**DOI:** 10.1186/1476-072X-12-5

**Published:** 2013-01-29

**Authors:** Xin Zhang, Penny A Cook, Paulo J Lisboa, Ian H Jarman, Mark A Bellis

**Affiliations:** 1 Liverpool John Moores University, Centre for Public Health, Henry Cotton Campus, Webster Street, Liverpool, UK; 2 University of Salford, School of Health Sciences, College of Health & Social Care, Allerton Building, Salford, UK; 3 Liverpool John Moores University, Department of Mathematics and Statistics, Liverpool, UK

**Keywords:** Area effect, Relative deprivation, Self-reported morbidity, Psychosocial pathway

## Abstract

**Background:**

Socioeconomic status gradients in health outcomes are well recognised and may operate in part through the psychological effect of observing disparities in affluence. At an area-level, we explored whether the deprivation differential between neighbouring areas influenced self-reported morbidity over and above the known effect of the deprivation of the area itself.

**Methods:**

Deprivation differentials between small areas (population size approximately 1,500) and their immediate neighbours were derived (from the Index of Multiple Deprivation (IMD)) for Lower Super Output Area (LSOA) in the whole of England (n=32482). Outcome variables were self-reported from the 2001 UK Census: the proportion of the population suffering Limiting Long-Term Illness (LLTI) and ‘not good health’. Linear regression was used to identify the effect of the deprivation differential on morbidity in different segments of the population, controlling for the absolute deprivation. The population was segmented using IMD tertiles and P^2^ People and Places geodemographic classification. P^2^ is a commercial market segmentation tool, which classifies small areas according to the characteristics of the population. The classifications range in deprivation, with the most affluent type being ‘Mature Oaks’ and the least being ‘Urban Challenge’.

**Results:**

Areas that were deprived compared to their immediate neighbours suffered higher rates of ‘not good health’ (β=0.312, p<0.001) and LLTI (β=0.278, p<0.001), after controlling for the deprivation of the area itself (‘not good health’—ß=0.655, p<0.001; LLTI—ß=0.548, p<0.001). The effect of the deprivation differential relative to the effect of deprivation was strongest in least deprived segments (e.g., for ‘not good health’, P^2^ segments ‘Mature Oaks’—β=0.638; ‘Rooted Households’—β=0.555).

**Conclusions:**

Living in an area that is surrounded by areas of greater affluence has a negative impact on health in England. A possible explanation for this phenomenon is that negative social comparisons between areas cause ill-health. This ‘psychosocial effect’ is greater still in least deprived segments of the population, supporting the notion that psychosocial effects become more important when material (absolute) deprivation is less relevant.

## Background

Socioeconomic status gradients for many health outcomes have been recognised in numerous studies [1-3]. Using both individual-level measures of deprivation and area-level (ecological) measures of deprivation, increased mortality, ill health indicators and reduced life expectancy are highly correlated with lower socioeconomic status [4-6]. While area-level measures of deprivation have been used as a proxy when individual measures of deprivation have been unavailable [7], there has been an increasing interest in the deprivation of the local neighbourhood effects *per se*, and there is now substantial evidence that neighbourhood deprivation influences health over and above the effect of individual deprivation [4,8-10]. Pickett and Pearl systematically reviewed the multilevel studies of neighbourhood effects on public health [4]. Most studies in their review confirmed the association between neighbourhood deprivation and poor health. The neighbourhood effect on health varies at different subgroups (e.g., males and female) [11], different types of areas (e.g., rural area and urban area)[12] and different geographical units [13].

There are two main interpretations for the explanation of the relatively poor health of people living in disadvantaged neighbourhoods: a psychosocial perspective and a neo-material perspective [14]. According to psychosocial theory [15,16], socioeconomic inequality increases an individual’s sense of being deprived of status, resulting in frustration, shame and stress, which in turn leads to adverse health consequences. Wilkinson [16] hypothesised that negative psychological effects caused physical ill-health through psycho-neuro-endocrine mechanisms. The term ‘psycho-neuro-endocrine’ refers to a biological pathway that links hormone fluctuations and human behaviour and mood disorders.

The neo-materialist theory suggests that those areas that are wealthier have more local facilities and resources and this has a positive impact on health [17,18]. In support of this, it has been demonstrated that people in less deprived areas acquire more collective material and social resources, including public services, recreation facilities, job opportunities and social support, which promotes health [19-21]. Pertinent to this paper, this theory leads to the prediction that even a relatively poor area may benefit from the effect of being located among less deprived areas because of better public services and facilities; this is the reverse of the prediction from the psychosocial theory, which would predict that surrounding wealth (relative to own) would be detrimental to health.

The analysis of deprivation and health data at a small geographical area gives the ideal opportunity to test these competing hypotheses. Relatively little research has been done using such ecological analysis, with those that do seeming to show conflicting findings. Cox et al. [22] aimed to determine whether the incidence of Type 2 diabetes in small areas in Tayside, Scotland (Statistical Output Areas, average population ~200) was associated with deprivation in neighbouring areas, after controlling for the deprivation of the area itself. The results supported the neo-materialistic interpretation, with type 2 diabetes more common in deprived areas, but lower in deprived areas that were surrounded by relatively less deprived areas. Allender et al.’s study [23] used small areas in the whole of England (wards, population ~6500), and measured relative deprivation for wards as the absolute difference between the deprivation of the ward and the average for all neighbouring wards. They found that higher inequality was associated with mortality from coronary heart disease. Although their study did not directly test the two hypotheses (since the direction of the inequality was not measured), they did interpret it as supporting the notion that inequalities are detrimental to overall population health.

One problem with interpreting some of these studies is that the deprivation of each area tends to be related to that of the areas around it, making it hard to investigate the unique contribution of the surrounding areas’ deprivation on a target locality’s population health. Previously, we demonstrated how to overcome this difficulty by generating an uncorrelated measure of deprivation inequality [24]. This was used to examine how neighbouring socioeconomic conditions influenced the mortality of a target locality, using small geographical units (the Lower Super Output Area (LSOA), average population ~1500) across the whole of England (n=32482). Areas that were surrounded by more affluent areas suffered greater mortality than those surrounded by areas of equal or lower affluence, which was consistent with the psychosocial model.

In this paper, we explore the association between the self-reported health status of an area and the deprivation differential between it and surrounding areas and compare the strengths of relationships with the mortality findings reported previously. Mortality and self-reported health status are usually considered to function similarly as indicators of population health. However, self-reported health can be considered to be a subjective evaluation that captures the full array of illnesses and symptoms of undiagnosed diseases in preclinical stages, and could be mediated by psychological status [25]. Since these measures may be more closely related to the proposed psychosocial explanation for health inequalities, we aim to test the psychosocial explanation of deprivation and health inequalities by comparing the health of areas where deprivation is high relative to their neighbours with those where deprivation levels are similar or less.

## Methods

### Measures of absolute and relative deprivation

Previously we have shown that, due to the strong association between an area’s deprivation and that of its neighbours, it is difficult to differentiate between the effects of the deprivation of the target area itself and that of its neighbours on a health outcome [24]. Here, we use two variables, derived from our previous paper, that represent an area’s absolute deprivation and the deprivation differential between an area and its immediately surrounding neighbours. The queen contiguity method was employed to define each Lower Super Output Area (LSOA) neighbour. The queen contiguity considers any area that shares a common boundary or vertex as a neighbour [26,27], which means all the surrounding areas are defined as neighbours. The deprivation measures were derived for each of England’s 32482 LSOAs, from the Index of Multiple Deprivation (IMD 2007, Department for Communities and Local Government). The original IMD for each LSOA and the weighted mean (Adjacent locality deprivation: ALD) of IMD scores of the surrounding LSOAs for each LSOA were entered in a principal components analysis to generate two uncorrelated (orthogonal) components, henceforth referred to as the ‘target area deprivation’ and the ‘deprivation differential’. The target area deprivation is the first principal component and is approximately equal to the sum of the two terms (PC1=0.76IMD+0.64ALD). The deprivation differential is the second component and represents the difference between the IMD score of the target area and the weighted mean of the IMD scores of the surrounding LSOAs (PC2=0.64IMD-0.76ALD) [24]. These derived variables have arbitrary values, with the target area deprivation ranging from −3.31 to 4.7, where a strongly positive value means the most deprived area. The deprivation differential ranges from −2.98 to 5.76, where strongly positive value means a big differential. See Zhang et al. [24] for full details and justification of this method.

### Self-reported morbidity

We used two morbidity indicators from the self-reported health questions in the 2001 UK census (extracted from Office for National Statistics (ONS) online data warehouse at http://neighbourhood.statistics.gov.uk/dissemination/ in February 2011). For the census question on general health all adult members of the population were asked whether, over the past 12 months, their ‘health had on the whole been good, fairly good, or not good?’. The census question on Limiting Long-Term Illness (LLTI) was ‘do you have any long term illness, health problem, or disability (including those due to old age) which limits your daily activities or the work you can do?’. The proportion of the population in each LSOA reporting LLTI and the proportion reporting that their general health in the preceding year was ‘not good’ were calculated. The denominator population was the number of adults aged 15–65 years in each LSOA.

### Data analysis

**Figure 1:**
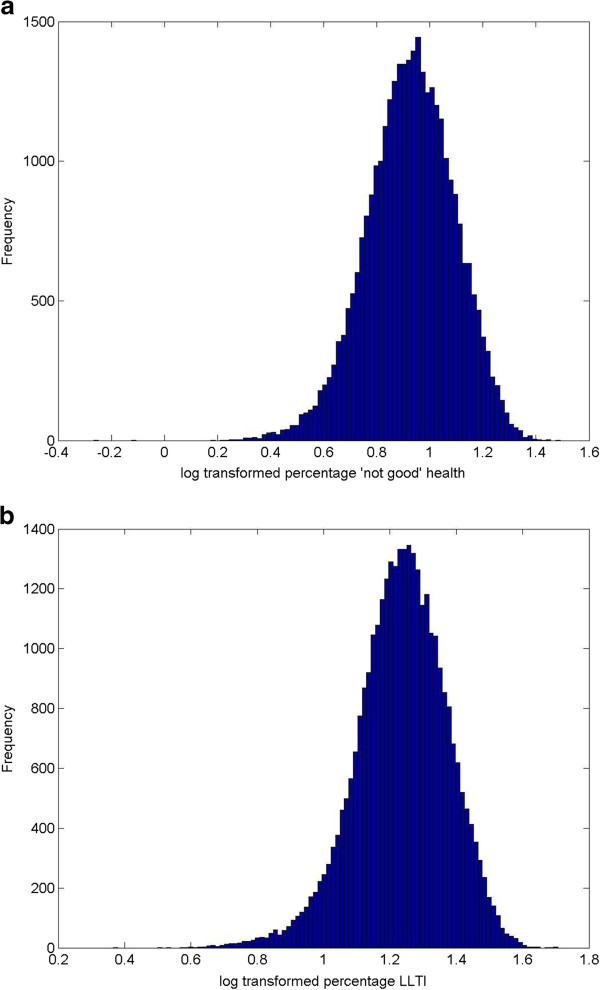
The distribution of the proportions of the population declaring a) ‘not good’ health (mean±SD, 0.92±0.16) and b) Limiting Long-Term Illness after log transformation, n=32482 small areas (mean±SD, 1.23±0.13).

Histograms for outcome variables were examined, and outcome data were log-transformed (logarithm base 10) to correct for moderately skewed distributions (Figure 1). Linear regression models were used to explore the ecological association between the predictor variables (target area deprivation and the deprivation differential) and the outcome variables (the proportion of the population declaring ‘not good health’ and the proportion declaring LLTI).

Further exploration of the data was undertaken by replicating the regression models within categories (thirds) of IMD and within classifications of a geodemographic population segmentation tool, P^2^ People and Places [28]. We used tertiles to divide the IMD data equally into three groups, each containing a third of overall data. The P^2^ segmentation subdivides a heterogeneous population into homogeneous subpopulations based on census and marketing and media survey data (the Target Group Index). P^2^ is used by the commercial sector and is compiled at the output area level (OA, population ~300). An LSOA level dataset is available for research, which was derived by aggregating the source data at LSOA level and assigning each LSOA to the nearest cluster centroid obtained from the OA level classification [28]. We present data for the ‘Tree’ level which identifies 13 neighbourhood types (see Appendix). P^2^ has been shown to perform well in analysis of health variables compared to other segmentation systems [29]. Regression analysis was performed in MATLAB 2011a.

## Results

**Figure 2:**
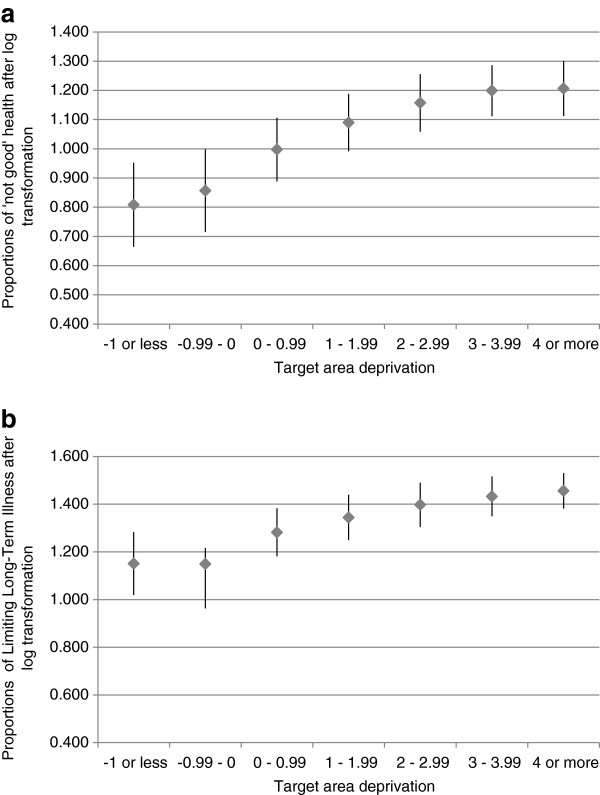
**Relationship between target area deprivation and (log transformed) proportions of a) not good health and b) Limiting Long-Term Illness, n=32482 small areas (means±standard deviations).** The target area deprivation is the Index of Multiple Deprivation 2007 (higher values define higher deprivation; units are arbitrary). The outcome variable is from the 2001 UK census for 32482 small areas across the whole of England.

**Figure 3:**
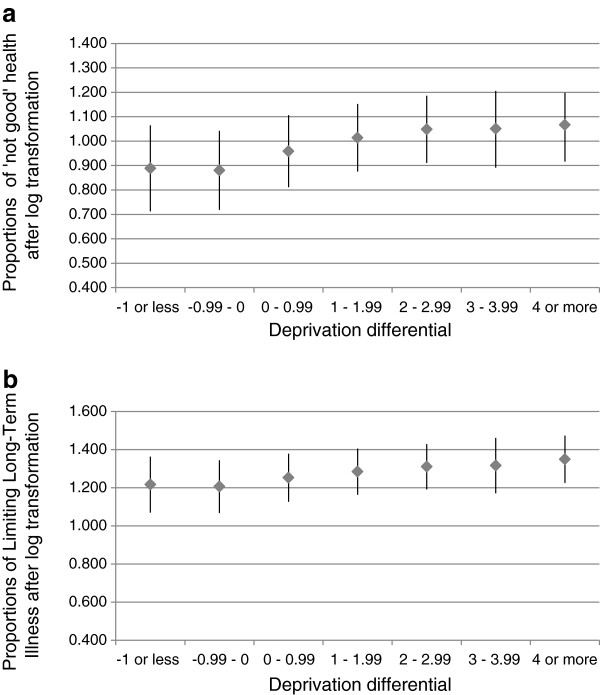
**Relationship between deprivation differential and (log transformed) percentages of the population declaring a) not good health and b) Limiting Long-Term Illness, n=32482 small areas (means±standard deviations).** The deprivation differential is calculated from the Index of Multiple Deprivation 2007 (higher values define higher deprivation; units are arbitrary). The outcome variable is from the 2001 UK census for 32482 small areas across the whole of England.

The proportions of ‘not good health’ and LLTI increased with increasing deprivation of the target area (Figure 2), indicative of the expected positive relationship between deprivation and morbidity indicators. Self-reported morbidity also increased the more deprived a target area was compared to its neighbours (i.e. as the deprivation differential increased: Figure 3).

Table 1 shows the bivariate and multivariate models to explain LLTI and ‘not good health’. Bivariate models looked at each outcome variable in turn and confirmed the relationships shown in Figures 2 and 3. To measure the extent to which the deprivation differential has an additional effect on morbidity, not explained by an area’s deprivation alone, an additive multivariate model was fitted to proportions of ‘not good health’ and LLTI (final column of Table 1). This multivariate model (with both the target area deprivation and the deprivation differential) explained 53% of the variation in ‘not good health’ and 41% of the variation in LLTI, and this explanatory power was greater than when either variable was considered separately. The slope of deprivation is approximately twice that of the deprivation differential, meaning that every one unit increase in deprivation was twice as harmful to health as a unit increase in the deprivation differential. However, although to a lesser extent, the effect of the deprivation differential was statistically significant (P<0.001).

**Table 1:** Regression models to explain the variation in percentage of the population declaring ‘not good health’ and limiting long-term illness

	**Bivariate regression model**	**Bivariate regression model**	**Multivariable regression model**
Not good health			
Target area deprivation Standardized slope (ß_1_ value)	0.655^***^	N/A	0.655^***^
Deprivation differential Standardized slope (ß_2_ value)	N/A	0.312^***^	0.312^***^
Adjusted R^2^	0.429	0.097	0.526
Limiting Long-Term Illness			
Target area deprivation Standardized slope (ß_1_ value)	0.548^***^	N/A	0.548^***^
Deprivation differential Standardized slope (ß_2_ value)	N/A	0.278^***^	0.278^***^
Adjusted R^2^	0.330	0.076	0.410

*** Significance level p< 0.001.

The predictor variables are target area deprivation and deprivation differential (derived from the Index of Multiple Deprivation 2007). The outcome variables are the log transformed percentages of the population declaring ‘not good’ health and the log transformed percentages of the population declaring LLTI (both from the 2001 UK census) for 32482 small areas across the whole of England. Two bivariate regression models regressed the target area deprivation and then the deprivation differential separately for each outcome variable. The multivariable regression model used two predictor variables together to predict each outcome variable.

The relative proportion of variation in morbidity explained by each deprivation measure varied between different population cohorts, as demonstrated using both the IMD thirds and People and Places segmentation (P^2^). Within each segment, the coefficients of the multivariate regression model for self-reported morbidity were re-estimated (Table 2 and 3). For both ‘not good health’ and LLTI, the beta coefficients for slope beta 1 (absolute deprivation) vary less across the IMD thirds than the beta 2 coefficients. Thus, the deprivation differential has a stronger effect in the least deprived third of LSOAs. The low beta 2 in the upper third suggests that the deprivation differential is not particularly important in the most deprived third. In fact, the effect of the deprivation differential on morbidity is approximately equal to that of the target area deprivation in the middle and least deprived thirds. Therefore, in least deprived areas, every unit increase in the deprivation differential was equally as harmful as a unit increase in the deprivation of the target area.

**Table 2:** Standardized β values for the relationship between deprivation and the percentage of the population declaring ‘not good health’ for two different data segmentations

**Data segmentation**	**Rank of deprivation**	**Slope β**_**1 **_**(target area deprivation)**	**Slope β**_**2 **_**(deprivation differential)**	**Ratio β**_**1**_**/β**_**2**_
IMD thirds segementation				
Upper third (most deprived)	1	0.531^***^	0.221^***^	2.202
Medium third (middle deprived)	2	0.555^***^	0.513^***^	1.081
Lower third (least deprived)	3	0.732^***^	0.748^***^	0.978
P^2^ categories segmentation				
New Starters	6	0.736^***^	0.237^***^	3.105
Urban Challenge	1	0.452^***^	0.200^***^	2.260
Multicultural Centres	3	0.604^***^	0.265^***^	2.279
Qualified Metropolitan	8	0.542^***^	0.246^***^	2.203
Weathered Communities	4	0.441^***^	0.252^***^	1.750
Urban Producers	5	0.627^***^	0.446^***^	1.405
Disadvantaged Households	2	0.701^***^	0.523^***^	1.340
Senior Neighbourhoods	9	0.541^***^	0.418^***^	1.294
Suburban Stability	7	0.534^***^	0.464^***^	1.150
Country Orchards	11	0.600^***^	0.577^***^	1.039
Rooted Households	10	0.555^***^	0.558^***^	1.008
Blossoming Families	12	0.601^***^	0.633^***^	0.949
Mature oaks	13	0.638^***^	0.688^***^	0.927

*** Significance level p< 0.001. The ranks of deprivation shows the order of deprivation for each cohort (13 = most affluent cohort, 1 = most deprived cohort).

Predictor variables are area deprivation and deprivation differential (Index of Multiple Deprivation 2007) and the outcome variable is the log transformed percentage of the population declaring ‘not good health’ (from the 2001 UK census) for 32482 small areas across the whole of England. The results (for two data segmentation-IMD thirds and People and Places P2) are ordered by decreasing dominance of the target area deprivation to the deprivation differential (ratio of the slopes, β1 to β2).

**Table 3:** **Table**2**Standardized β values for the relationship between deprivation and the percentage of the population declaring Limiting Long-Term Illness (LLTI) for two different data segmentations**

**Data segmentation**	**Rank of deprivation**	**Slope β**_**1 **_**(target area deprivation)**	**Slope β**_**2 **_**(deprivation differential)**	**Ratio β**_**1**_**/β**_**2**_
IMD thirds segementation				
Upper third (most deprived)	1	0.409^***^	0.112^***^	3.651
Medium third (middle deprived)	2	0.336^***^	0.298^***^	1.127
Lower third (least deprived)	3	0.577^***^	0.602^***^	0.958
P2 categories segmentation				
Urban Challenge	1	0.409^***^	0.055^***^	7.436
Qualified Metropolitan	8	0.400^***^	0.056^***^	7.142
New Starters	6	0.659^***^	0.172^***^	3.831
Multicultural Centres	3	0.537^***^	0.179^***^	3.000
Weathered Communities	4	0.366^***^	0.158^***^	2.316
Urban Producers	5	0.643^***^	0.397^***^	1.619
Disadvantaged Households	2	0.706^***^	0.500^***^	1.412
Senior Neighbourhoods	9	0.386^***^	0.302^***^	1.278
Suburban Stability	7	0.426^***^	0.354^***^	1.203
Rooted Households	10	0.436^***^	0.404^***^	1.079
Country Orchards	11	0.567^***^	0.546^***^	1.038
Blossoming Families	12	0.550^***^	0.559^***^	0.983
Mature oaks	13	0.567^***^	0.606^***^	0.935

*** Significance level p< 0.001. The ranks of deprivation shows the order of deprivation for each cohort (13 = most affluent cohort, 1 = most deprived cohort).

Predictor variables are area deprivation and deprivation differential (Index of Multiple Deprivation 2007) and the outcome variable is log transformed percentages of the population declaring LLTI (from the 2001 UK census) for 32482 small areas across the whole of England. The results (for two data segmentation-IMD thirds and People and Places P2) are ordered by decreasing dominance of the target area deprivation to the deprivation differential (ratio of the slopes, β1 to β2).

The P^2^ segments are ordered by decreasing proportion of the effect of target area’s deprivation (the first slope, or beta value, β_1_) over the effect of the deprivation differential (β_2_), such that those with the highest dominance of the target area deprivation are presented near the top and segments where the effects of the deprivation differential and deprivation were equal are near the bottom (Tables 2 and 3). The order of the segments was similar for both proportions of ‘not good health’ (Table 2) and LLTI (Table 3). This analysis again demonstrates a stronger effect of the deprivation differential in the relatively more affluent categories (Mature Oaks, Blossoming Families, Country Orchards, Rooted Households). In contrast, the relatively deprived categories (Urban Challenge, Disadvantaged Households and Multicultural Centres) did not follow any particular order with reference to the relative effect of the deprivation differential. Instead, New Starters and Qualified Metropolitans, which were mediumly deprived cohorts, were strongly influenced by target area’s own deprivation.

## Discussion

Here we present data for the whole of England that demonstrate that socioeconomic inequality between neighbourhoods leads to poorer health: the population of a small area (comprising a population of approximately 1500 persons, n=32482) suffered greater ill-health if it was surrounded by areas of lower deprivation. We further demonstrate that these links are stonger than the previously described relationship between neighbourhood inequality and mortality, in a study that used the same methodology [24]. Allender et al. carried out an analysis using larger geographical areas within England (wards: population ~6500, n=7927) [23], and showed that deprivation inequality was harmful for population health (as measured by rates of mortality from coronary heart disease). However, relative deprivation had a relatively weak effect and did not improve the predictive power of their models. Our study confirmed the association at relatively smaller areas and also identified the direction of the influence of the deprivation differential on health. In addition, we found that the deprivation differential (relative deprivation) did improve the predictive power across the whole dataset (e.g. from 43% to 53% for ‘not good health’).

The fact that deprivation inequality is linked to ill-health has been explained by two competing hypotheses, which have generated much controversy [15,30]. The two hypotheses provided two distinct predictions for the direction of the relationship between neighbourhood inequalities and health. We did not find support for the neo-materialistic hypothesis, which predicted that poorer areas surrounded by greater affluence would have improved health (as a result of better infrastructure). The psychosocial model [16] predicts poorer health in a more deprived area surrounded by relatively less deprivation than would be found if the same area was surrounded by equivalent deprivation: which was indeed what our results have shown. Socioeconomic inequality is hypothesised to have a psychological and emotional impact which can lead to deterioration in physical health. The proponents of the psychosocial model have articulated a plausible biological pathway: the response to psychological stress involves the release of hormones (e.g. glucocorticoids) by the neural nerve and endocrine system, which circulate in blood system [31]. This stress response is beneficial in the short-term (e.g., glucocorticoid secretion promotes the metabolism of protein and lipids to carbohydrates to give the body energy). However, the long-term secretion of hormones under psychological stress (i.e., glucocorticoid excess) leads to hypertension (high blood pressure) and cardiovascular disease [32]. Here we suggest that social comparison is not only harmful to health in a wider social context, but it also happens between neighbourhoods.

We previously demonstrated the same between-area inequality effect using the more objective measure of mortality [24]. We argue that the two self-reported health variables used in this study could represent an intermediate step between psychosocial stress and an objective adverse outcome such as mortality, supported by the fact that self-reported health was influenced to a greater extent by the surrounding neighbourhood deprivation than was mortality. For mortality, when the data were segmented, the effect of the target area deprivation was larger in every population cohort (most deprived third: 2.5 times greater; middle deprived: 2 times greater), with the effect of the deprivation differential approaching equality within the least deprived cohorts (least deprived: 1.2 times greater) [24]. In this paper we show that the difference in deprivation between one area and its neighbours has an equally strong effect on self-reported health as the deprivation of the area itself in the middle third and the least deprived third of the areas. This observation fits with the notion of poor self-reported health representing an intermediate (and more senstive) response to relative deprivation.

The fact that both mortality and morbidity show similar relationships with the target area deprivation and the deprivation differential is not surprising as self-reported morbidity is highly correlated with mortality: for example, for England as a whole, there is a strong correlation between self-reported ‘not good health’ and all-cause mortality (r=0.86, n=352 English local authorities) [33]. Measures of self-reported health are also strongly related to objective measures of morbidity [34,35]. It has also been shown that a response of ‘good health’ on this single question is more strongly linked to physical health than to the mental or social health domains of the SF-36 health survey [36]. However, it is also plausible that such self-assessment could additionally be influenced by psychological factors. If a relatively affluent area is situated within a wider area that is even more affluent, then individuals might feel less positively about their status and possibly also their health than they would if they lived in an area surrounded by equivalent affluence. In this case, psychosocial effects may particularly influence the perception of self-rated health compared to other, less subjective, measures of health. Although self-reported health can be criticised for being too subjective [33,37], it is precisely this element of subjectivity that might explain the stronger effect of relative deprivation on self-reported health status shown in this paper compared to the previously demonstrated relationship with mortality [24]. This is consistent with the notion that psychosocial effects mediate the relationship between health and the deprivation of surrounding areas.

Areas at the less deprived end of the spectrum have health that is better on average. However, there is likely to be a non-linear relationship between deprivation and health such that reductions in deprivation have less impact on health for the least deprived areas [38]. Thus, at the lower end of the deprivation spectrum (i.e. in more affluent areas) there may be more capacity for average health at an area level to vary in response to a source of inequality that is relatively removed (i.e. comparisons between neighbouring areas of population size 1500, with an average distance between them of 6 km). In the more deprived areas, health is already depressed by the poverty of the area itself. Moreover, the psychological effects of relative deprivation (or relative affluence) may differ depending on whether a person is poor or affluent. There is some evidence to support this: affluent people living in poorer areas rated themselves as higher on the social ladder than equally affluent people living in affluent neighbourhoods, while poorer people living in affluent areas rated themselves more highly than equally poor people living in poor areas [39].

Interestingly, the only other study that we could find that uses a similar methodology [22] finds the reverse relationship between deprivation differential and ill-health, in this case the incidence of type 2 diabetes. Cox et al.’s study was also set up as a test of the two hypotheses, and therefore found support for the neo-materialistic approach. However, their study used smaller geographical areas (Output areas, around 150 households). The outcome measure used (diabetes) relied on a diagnostic resource that may have been more available in surrounding wealthier areas, and it is not known to what extent their findings reflect the underlying incidence of diabetes or the likelihood of diagnosing the condition. Neither the outcome measures used in this study (self-reported ill-health derived from the census) nor our previous study (using routine data on mortality) relied on the availability of any services for diagnosis.

For the affluent P^2^ People & Places categories of ‘Mature Oaks’, ‘Country Orchards’, ‘Blossoming Families’ and ‘Rooted Households’, a one unit increase in the deprivation differential was as significant for health as a unit increase in the deprivation of the area itself. Mature Oaks and Country Orchards are relatively wealthy, with a predominance of retired couples, while Blossoming Families tend to be composed of younger families with children (see appendix for description of P^2^ People & Places classification). In contrast, at the other end of the categorisation, the effect of the deprivation differential on self-reported health status did not always follow the order of average deprivation of the groups. The medium deprived groups, New Starters and Qualified Metropolitans, showed the weakest (but still significant) effect of the deprivation differential. This finding was similar to that observed for mortality [24]. Previous research has shown that New Starters and Qualified Metropolitans are outliers in several analyses of ill-health and have more risk indicators than would be predicted by the deprivation alone [40]. New Starters have higher rates of smoking-related and alcohol-related conditions, mental health conditions and all cause mortality. In contrast, Qualified Metropolitans have lower than expected rates across many indicators (e.g. accidents, asthma, coronary heart disease). The features of these groups that make them outliers across a range of other indicators may be the same as those that lead to them having a weaker relationship with the deprivation differential than would be expected. However, the mechanism of this is unknown.

Several limitations to this study should be acknowledged. First, the deprivation indices and health indicators were generated from census data and then applied at an aggregate level, which raises the possibility of the ‘ecological fallacy’ whereby the average characteristics of the population are assumed to represent the individial [41,42]. This pooling of populations for analysis would tend to towards the null rather than to spurious significance. Second, the morbidity data (‘not good health’ and LLTI) used the readily available public dataset and were not standardised by age. However, self-reported health differs by age, for example, old people are more likely to report LLTI [43,44]. Thus, the evaluation of deprivation differential on morbidity still needs to be further validated. However, our previous study using mortality as the outcome variable did use age-standardised data and found a similar relationship between the deprivation differential and mortality [24], suggesting that the patterns are robust. Third, we were unable to take migration into account, which might bias our results. It is known that healthy, affluent people are more likely to move away from less favourable environments [45]. In a Dutch study, those with higher education levels were prone to move out of relatively poor areas [46]. In our study, the morbidity data were collected in 2001, while The IMD index was released in 2007 (using data from 2001 to 2005). It is possible that migration caused the population to change between the two time points. Such migration could influence the illness rate and deprivation status of the origins and destinations, and then confound the relationship between health and deprivation [47,48]. However, for this to bias the results in favour of the observed deprivation differential effect, the migration would have to happen at a greater rate across borders with a higher deprivation differential. Migration effects may be more likely to bias the results towards the null.

## Conclusions

We carried out a national analysis on small-area morbidity data that has shown that greater inequality between neighbouring areas leads to poorer health, and that the impact of inequality is especially marked in least deprived areas. As Wilkinson and Marmot have argued, once a nation passes through the ‘epidemiological transition’, absolute deprivation might not be the fundamental determinant of health; deprivation inequality (deprivation differential) might play a significant role for population health [16]. Our findings on mortality data (previously [24]) and self-reported morbidity data (here) extend the influence of deprivation inequality on health to the small are level (LSOAs) for the whole of England. By comparing the results on mortality and self-reported morbidity, this study suggested that self-reported morbidity measures, which embrace wellbeing as well as a wide variety of possible health problems, appear particularly sensitive to differentials in deprivation, and may be more closely related to the psychosocial determinants of health.

## Appendix

See Table 4 for description of P2 People & Places classification.

**Table 4:** P^2^ People & Places geodemographic people classification description [28]

**P**^**2 **^**categories**	**Description**
Mature Oaks	Mature Oaks are generally middle-aged and older people, with many aged 45 to 64 and past retirement age. The majority are married couples with teenage children still living with them, or grown up children who have left home. Jews and Protestants are common in this Tree.
Country Orchards	Most members of this Tree are aged 55 to 65, with many being past retirement age but few being older than 75. They tend to be married couples whose children have left home, although there are still some children in the younger households.
Blossoming Families	This Tree is mainly made up of families, often aged 25 to 54 who are either married or cohabiting. There are many infants and young children and some teenagers.
Rooted Households	This Tree is generally an older group but has a wide spread of age groups, ranging from young adults to those of pension age. Most are married couples and few have children living at home. They generally originate from the UK and most are Christians, with many in Northern Ireland being Protestants.
Qualified Metropolitans	This Tree is mainly made up of young adults, aged 16 to 35 who are cohabiting and do not have children. A large number are students and there are some single-person households. There is also a multicultural population.
Senior Neighbourhoods	Most members of this Tree are retired, aged 55 to 75 and over with a significant number being over 74, although some are late middle-aged. There are very few children and many people live on their own.
Suburban Stability	This Tree covers an extremely wide range of age groups, from young families with children right up to those over 75 years old. Many of the parents are unmarried.
New Starters	This Tree consists mainly of young people aged 16 to 34 with no children. There are a lot of students and people living alone. Some older households, aged 35 to 54, do have children but few of the couples are married, choosing to cohabit instead. There is also a mix of people from multicultural backgrounds.
Multicultural Centres	This Tree consists mainly of families, some of which are large, who originate from India, Pakistan, Bangladesh or Africa with a good proportion from the Caribbean and China. There is a combination of young parents with children and older parents with teenagers. The majority are Muslims or Jews and although the parents were born outside the UK, their children have been born here.
Urban Producers	This Tree has a high proportion of lone-parent families. Many households are couples, aged 25 to 34, who are unmarried and have children. There are also some people aged 16 to 24 with children.
Weathered Communities	Most of this Tree are past retirement age with many being older than 75 and living alone.
Disadvantaged Households	This is generally a young Tree, mainly made up of young parent families who are aged 16 to 34 and have young children. The proportion of married couples is relatively low, with many families being cohabiting couples or lone-parents.
Urban Challenge	The majority of people in this Tree are elderly. Some are over 75 and a high proportion live alone.

## Abbreviations

LLTI: Limiting Long-Term Illness; IMD: Index of Multiple Deprivation; LSOA: Lower Super Output Area; ONS: Office for National Statistics; P2: People and Places segmentation.

## Competing interests

The authors declare that they have no competing interests.

## Authors’ contributions

XZ, PAC, PJL, IHJ developed the area effect on health outcome project and executed the studies. PAC, PJL, IHJ and MAB provided oversight and advice for the design and interpretation of the statistical analyses. XZ carried out the statistical analysis. XZ and PAC drafted the manuscript. All authors contributed to the interpretation of findings, the writing of the paper and approved the final draft.
